# Programmed Death-Ligand 1 Expression in Triple-Negative Breast Cancer: Insights from a Mexican Cohort

**DOI:** 10.3390/cancers18142182

**Published:** 2026-07-08

**Authors:** Cynthia Villarreal-Garza, César Octavio Lara-Torres, Jesus Edgardo Hernandez-Hernandez, Daniela Vázquez Juárez, Gabriela Sofía Gómez-Macías, Paula Cabrera-Galeana, Fany Iris Porras-Reyes, Víctor Manuel Pérez-Sánchez, Antonio Nateras-Pérez, Gabriela Lugo-Martinez, Alejandro Aranda-Gutierrez, Alejandro Mohar

**Affiliations:** 1 Oncology Institute and Breast Cancer Center, Hospital Zambrano Hellion TecSalud, Tecnologico de Monterrey, San Pedro Garza Garcia 66260, Mexicoalejandroarandag@hotmail.com (A.A.-G.); 2 Subdirección de Patología, Instituto Nacional de Cancerología, Mexico City 14080, Mexico; 3 Escuela de Medicina y Ciencias de la Salud, Tecnologico de Monterrey, Monterrey 64710, Mexico; a00832501@tec.mx (J.E.H.-H.);; 4 Facultad de Medicina del Hospital Universitario, Universidad Autónoma de Nuevo León, Monterrey 64460, Mexico; 5 Subdirección de Oncología y Hematología, Instituto Nacional de Cancerología, Mexico City 14080, Mexico; 6 MSD, Mexico City 01090, Mexico; 7 Unidad de Investigación Biomédica en Cáncer, Instituto Nacional de Cancerología-Instituto de Investigaciones Biomédicas, Universidad Nacional Autónoma de México (UNAM), Mexico City 14080, Mexico

**Keywords:** triple-negative breast cancer, biomarker, CPS, PD-L1, TILs, Mexico

## Abstract

**Background**: Pembrolizumab-containing regimens have become the standard of care across the spectrum of triple-negative breast cancer (TNBC). While their use in the neoadjuvant setting is independent of biomarker status, their application in metastatic disease remains strictly contingent upon PD-L1 expression. Given that PD-L1 prevalence can vary significantly by ethnicity and geography, the lack of specific data for the Mexican population creates a challenge for optimizing treatment in the metastatic setting. This study sought to characterize PD-L1 positivity rates in a Mexican TNBC cohort to better define the local molecular landscape. **Methods**: We conducted a retrospective study across two cancer centers in Mexico to assess PD-L1 positivity in a cohort of women with TNBC (stages I–IV) diagnosed between 2006 and 2021. PD-L1 expression was assessed and evaluated centrally using the 22C3 pharmDx assay, with a Combined Positive Score (CPS) of ≥1 considered positive. We explored the association between PD-L1 expression and clinicopathological features. **Results**: Of the 298 TNBC patients identified, 285 (96%) had sufficient tissue for CPS evaluation and thus were included in the analysis. PD-L1 positivity was observed in 29.1% of the cohort, and 13.3% of patients had a CPS ≥ 10. PD-L1 positivity was associated with higher histological grades (91.3% vs. 78.5%, *p* = 0.035) and TILs ≥ 30% (22.2% vs. 10.0%, *p* = 0.007). Additionally, pre-treatment surgical specimens were more frequently PD-L1 positive than tumor biopsies (56.6% vs. 30.7%, *p* < 0.001). **Conclusions**: This study characterizes the PD-L1 landscape in Mexican women with TNBC, reporting a 29.1% prevalence of CPS ≥ 1. The strong association between PD-L1 positivity and high TILs/histological grade highlights the role of the immune microenvironment in these aggressive phenotypes. Given the significant variability observed between specimens (biopsy vs. surgical), clinicians should consider the dynamic nature of PD-L1 expression when choosing treatment strategies.

## Simple Summary

Immunotherapy using pembrolizumab has transformed triple-negative breast cancer (TNBC) care, improving survival regardless of biomarker status in early-stage disease, but remaining restricted to patients with a Programmed Death Ligand 1 (PD-L1) Combined Positive Score (CPS) ≥ 10 in the first-line metastatic setting. However, real-world PD-L1 prevalence remains poorly characterized in Hispanic populations from Latin America. This study evaluated PD-L1 expression in a 15-year cohort of 285 Mexican women with TNBC using the standard 22C3 pharmDx assay. We found an overall positivity rate (CPS ≥ 1) of 29.1%, while only 13.3% reached the metastatic therapeutic threshold of CPS ≥ 10. Notably, pre-treatment surgical specimens demonstrated significantly higher CPS values than core needle biopsies, a discrepancy probably driven by intratumoral heterogeneity and tissue sampling volume. Furthermore, PD-L1 positivity was strongly associated with advanced histological grades and highly immunogenic microenvironments (stromal tumor-infiltrating lymphocytes ≥ 30%). These findings establish a vital regional biomarker baseline and support a practical clinical strategy for resource-limited settings: when available, testing on larger pre-treatment surgical resections should be considered to prevent underestimating treatment eligibility.

## 1. Introduction

In the complex landscape of breast cancer, triple-negative breast cancer (TNBC) is notable for its aggressive clinicopathological features and inferior outcomes compared to other subtypes, positioning it as a central focus for innovative treatment strategies [1]. For many years, the standard of care for early and locally advanced TNBC was neoadjuvant chemotherapy, with adjuvant capecitabine or olaparib added in cases of residual disease [2,3]. Similarly, chemotherapy was the standard treatment for advanced TNBC, with olaparib and talazoparib as additional options for patients carrying *BRCA* pathogenic variants [4,5]. Fortunately, the treatment paradigms for both early and advanced TNBC have shifted with the introduction of immunotherapy to clinical practice.

In the early and locally advanced settings, the KEYNOTE-522 trial changed the standard of care by demonstrating that chemoimmunotherapy with pembrolizumab was superior to traditional chemotherapy for treating high-risk disease [6]. This shift was further reinforced by the trial’s 5-year overall survival (OS) data, showing an absolute benefit of 4.9% with the addition of pembrolizumab [7]. For advanced TNBC, the KEYNOTE-355 trial demonstrated that adding pembrolizumab to chemotherapy (whether nab-paclitaxel, paclitaxel, or gemcitabine plus carboplatin) significantly prolonged both progression-free survival and OS in the first-line setting [8]. As a result, pembrolizumab has become an integral part of the standard of care across the continuum of TNBC.

While pembrolizumab has proven advantageous in treating TNBC, accurately predicting which patients will benefit most from it remains a challenge. In the early TNBC setting, pembrolizumab is approved regardless of programmed death ligand 1 (PD-L1) expression [6]. However, in the advanced setting, its approval is restricted to cases with a Combined Positive Score (CPS) of at least 10 [8]. This distinction suggests that while PD-L1 expression may offer some insight into the efficacy of immunotherapy, it is not a definitive biomarker, underscoring the need for further research to improve patient selection.

Moreover, real-world observational studies reveal substantial variability in PD-L1 positivity rates (defined as a CPS ≥ 1 by the 22C3 pharmDx assay), ranging from 32.4% to 53.9% [6,8,9,10,11,12]. These rates are lower than those reported in key immunotherapy TNBC clinical trials, further complicating the use of PD-L1 as a reliable predictor of treatment response. Therefore, understanding the variability in PD-L1 expression across different populations is crucial. Notably, there are limited data on PD-L1 expression rates within the Mexican population, despite TNBC accounting for 16–23% of all breast cancers in Mexico and Mexican patients being included in the KEYNOTE-355 and IMpassion130 trials [13].

This study aims to evaluate the PD-L1 positivity rate in a cohort of Mexican women with TNBC and identify factors associated with positive CPS values. By doing so, we aim to provide the local evidence base necessary to optimize patient selection for immunotherapy and refine precision medicine strategies for Mexican women with TNBC.

## 2. Materials and Methods

### 2.1. Study Setting and Participants

We conducted a retrospective study at two Mexican centers: the Instituto Nacional de Cancerologia (INCan) in Mexico City and the Breast Cancer Center at TecSalud in Nuevo Leon, Mexico. INCan is one of the thirteen National Health Institutes within the public Mexican health system and serves as its primary cancer referral center. TecSalud is an academic and private hospital affiliated with the School of Medicine and Health Sciences of Tecnologico de Monterrey.

Eligible participants were women aged 18 and older with a diagnosis of TNBC at stages I–IV, diagnosed between 2006 and 2021, and who had formalin-fixed paraffin-embedded tumor tissue blocks for CPS evaluation. Tissue samples could be from either pre-treatment breast biopsies or pre-treatment breast surgical specimens.

**Figure 1:**
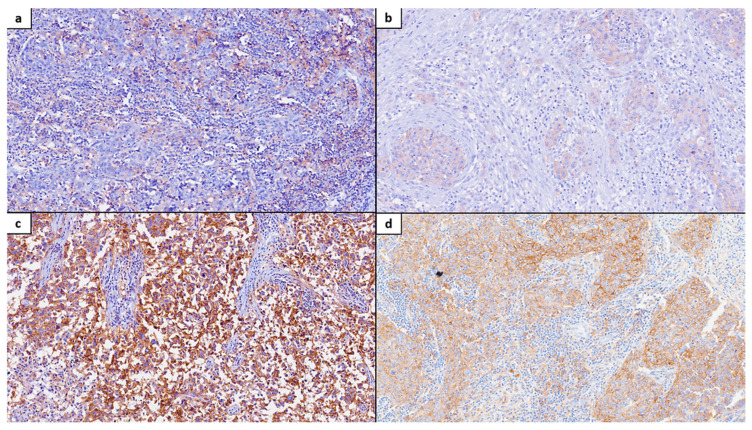
CPS evaluation (microscope magnification 20×) of PD-L1 expression in TNBC in Mexican patients using the 22C3 pharmDx assay. (**a**) CPS 10, (**b**) CPS 35, (**c**) CPS 50, (**d**) CPS 200.

PD-L1 expression was assessed centrally using the 22C3 pharmDx assay (Agilent Technologies, Inc., Santa Clara, CA, USA) (Figure 1), with positivity defined as a CPS of at least 1. To account for potential variations in pathology practices, diagnostic criteria, and technology across the 15-year inclusion period, all specimens underwent centralized re-evaluation. TNBC diagnosis was confirmed retrospectively based on negative estrogen receptor (ER) and progesterone receptor status (defined as less than 1% nuclear staining by immunohistochemistry (IHC)) and HER2 overexpression status (0 or 1+ by IHC, or 2+ by IHC without amplification by fluorescence in situ hybridization).

Tumor-infiltrating lymphocytes (TILs) were evaluated as a continuous variable on a single hematoxylin and eosin-stained section of the primary tumor sample, following the internationally accepted guidelines established by the International Immuno-Oncology Biomarker Working Group on Breast Cancer. All TIL assessments were performed independently by two specialized breast pathologists who were blinded to the clinical outcomes and the final combined positive score results. In cases of numerical discrepancy greater than ten percent between the two evaluators, a consensus score was reached using a multi-head discussion microscope.

Exclusion criteria were technical tissue inadequacy for PD-L1 assessment and missing clinical records. Specifically, samples were excluded from the final analysis if the central pathologist determined they did not meet assay requirements, including fewer than 100 viable tumor cells on the PD-L1 slide or presenting with non-specific background staining of ≥1+ intensity that obscured specific staining.

### 2.2. Statistical Analysis

Statistical analyses were performed using IBM SPSS Statistics Version 25.0 (IBM Corp., Armonk, NY, USA). Patients were categorized based on their PD-L1 status (positive vs. negative), with analyses performed for CPS ≥ 1 and CPS ≥ 10 groups. Descriptive statistics were used to summarize categorical variables as frequencies and proportions, and quantitative variables as medians and ranges. χ^2^ tests, Fisher’s exact tests, and *t*-tests were applied to assess differences between groups, as appropriate. Statistical significance was defined as a two-sided *p*-value of <0.05.

### 2.3. Ethics Statement

This study was conducted following the Declaration of Helsinki and approved by the Research and Ethics Committee of both participating institutions.

## 3. Results

### 3.1. Patients’ Demographics and Clinicopathological Features

A total of 298 patients with TNBC were identified, with 241 (81%) from INCan and 57 (19%) from TecSalud. After analyzing the available tissue, 285 (96%) patients had samples with sufficient cellularity for adequate CPS evaluation and were included in the final analysis. In the entire cohort, the PD-L1 positivity (CPS ≥ 1) rate was 29.1%, and 13.3% of patients had tissues with a CPS ≥ 10. Notably, none of the patients in this cohort received pembrolizumab, as the last patients included were diagnosed in 2021, before it was widely available in Mexico.

### 3.2. Factors Associated with PD-L1 Positivity

The clinicopathological features based on PD-L1 status are summarized in Table 1. Patients in the CPS ≥ 1 group were more likely to have higher histological grades (91.3% vs. 78.5%; *p* = 0.035) and had a higher likelihood of exhibiting ≥30% TILs (22.2% vs. 10.0%; *p* = 0.007). Additionally, pre-treatment surgical specimens were more frequently PD-L1 positive than pre-treatment tumor biopsies (56.6% vs. 30.7%, *p* < 0.001). No significant differences were observed regarding age at diagnosis, obesity status, histological type, Ki-67 indices, time elapsed between biopsy/surgery and CPS evaluation, lymph node status, clinical stage at diagnosis, or pathological complete response rates.

Patients in the CPS ≥ 10 group were younger at the time of TNBC diagnosis (44 vs. 50 years; *p* = 0.008) and had a higher percentage of TILs (≥30%; 33.3% vs. 10.6%; *p* < 0.001). Pre-treatment surgical specimens were also more frequently CPS ≥ 10 than pre-treatment tumor biopsies (68.4% vs. 33.6%; *p* < 0.001). No significant differences were observed in other parameters.

## 4. Discussion

In this study, we observed a PD-L1 positivity (CPS ≥ 1) rate of 29.1% among a cohort of Mexican women with TNBC, with 13.3% of analyzed samples also exhibiting a CPS ≥ 10. We utilized a CPS of ≥1 as our primary threshold for defining PD-L1 positivity to thoroughly characterize the baseline immune-active landscape of our cohort, as this cutoff is widely recognized as the biological benchmark for low-level immune checkpoint expression in TNBC clinical trials [6]. However, clinical decision-making for first-line pembrolizumab in the advanced setting is strictly restricted to patients exhibiting a CPS of ≥10, based on the survival benefit demonstrated in the KEYNOTE-355 trial [8]. This creates a critical distinction between biological positivity and therapeutic eligibility. While nearly one-third of our cohort, representing 29.1%, demonstrated an immune-positive microenvironment with a CPS of ≥1, only 13.3% met the strict threshold required for metastatic treatment. This narrower eligibility underscores a major challenge in real-world clinical practice, particularly in middle-income countries where access to regular tissue re-testing and high-cost immunotherapy is already constrained. Relying solely on initial primary tumor biopsies, which we found exhibited significantly lower rates of a CPS of ≥10 compared to surgical specimens, risks underestimating therapeutic eligibility and further widening the treatment access gap for patients who might otherwise derive a significant survival benefit from checkpoint inhibition.

Notably, our findings differ from the PD-L1 positivity rates reported in several major TNBC immunotherapy clinical trials, including 40.9% in IMpassion130, 45.0% in IMpassion131, 75.1% in KEYNOTE-355, 82.9% in KEYNOTE-522, and 87.0% in GeparNuevo [6,8,12,14,15]. In addition, the KEYNOTE-355 study reported a CPS ≥ 10 rate of 38.1%, which is also significantly higher than the 13.3% rate observed in our study [8].

Differences between the PD-L1 positivity rates observed in our cohort and those reported in major clinical trials may be explained by a combination of ethnic, biological, and methodological factors. Ethnic variation may influence the tumor immune microenvironment and the distribution of PD-L1 expression across populations [16]. Biological differences, including tumor size, grade, immune cell infiltration, and intratumoral heterogeneity, may also contribute to variability in PD-L1 prevalence [17,18,19]. Methodological factors are equally important, as PD-L1 results can be affected by specimen type, timing of tissue sampling, pre-analytic conditions, antibody clone, assay platform, and scoring algorithm. Therefore, cross-study comparisons should be interpreted cautiously, particularly when differences exist in patient population, tissue source, and PD-L1 testing methodology. Notably, similar to our study, previous real-world data have shown lower 22C3-defined PD-L1 positivity rates in TNBC, ranging from 32.4% to 53.9% [6,8,9,10,11,12]. These variations reinforce the inconsistency in PD-L1 expression across different cohorts and populations and confirm that many factors influence it.

Moreover, a major consideration in studies spanning multiple decades is whether historical changes in tissue processing, prolonged storage, or specimen degradation over time could artifactually alter biomarker expression. To mitigate these variables, all archival blocks in our cohort underwent centralized processing and uniform immunohistochemical staining using the standardized 22C3 pharmDx assay clone, eliminating historical variations in testing platforms, antibody selection, or subjective scoring systems. Regarding long-term storage, the available literature provides conflicting data on whether the chronological age of archival blocks compromises antigen stability. In our cohort, we statistically verified that the time elapsed between original tissue collection and the current CPS evaluation was not significantly associated with lower rates of PD-L1 positivity, suggesting stable antigen preservation. However, we acknowledge that conflicting findings have been reported in the recent literature; specifically, a retrospective study evaluating TNBC archival specimens found that prolonged storage, particularly beyond three years, led to a significant decline in 22C3-defined PD-L1 immunoreactivity and a higher risk of false-negative CPS results [20]. These discrepancies across cohorts underscore that while proper processing can maintain baseline stability, storage duration remains a potentially critical pre-analytical variable that must be carefully interpreted when evaluating archival diagnostic tissues in real-world clinical practice.

Importantly, our results revealed a significant association between higher histological grades and PD-L1 positivity, as observed in previous studies [21,22]. Evidence suggests that aggressive tumor characteristics, such as lymph node metastases, high histological grades, and ER-negativity, are linked to more robust immune responses and, thus, higher PD-L1 expression rates [21]. Consistent with this, we also found that a higher percentage of TILs (≥30%) was significantly associated with higher CPS values, highlighting the crucial role of the immune microenvironment in determining PD-L1 expression. This relationship is particularly relevant in TNBC, where TILs have been linked to improved responses to immunotherapy [23,24].

Furthermore, PD-L1 expression is also influenced by the type of tissue in which the CPS is evaluated (i.e., primary breast tumor vs. metastatic site) [25]. For example, post hoc analyses from the IMpassion130 trial (which allowed patient enrollment based on biopsies from both primary breast tumors and metastatic sites) revealed that the PD-L1 positivity rate was higher in the primary tumors (44.0%) compared to metastatic sites (36.0%) [26]. In alignment with this, the KEYNOTE-522 and GeparNuevo trials, which focused on early-stage disease, reported higher PD-L1 positivity rates than other TNBC immunotherapy trials [6,15]. This is likely because, as a tumor progresses, immunosurveillance mechanisms diminish or are lost, resulting in immune evasion and accounting for the lower PD-L1 positivity rates observed in metastatic disease. Notably, the IMpassion130 post hoc analysis also showed that PD-L1 positivity rates varied across different TNBC disease sites: 13% in liver, 30% in soft tissue, 43% in breast tissue, 43% in lung, 48% in skin, 51% in lymph nodes, and 36% in other sites [26]. Consequently, the lower PD-L1 expression in metastatic tissues may be due to reduced positivity rates in specific sites rather than an overall decrease in PD-L1 positivity across all metastatic locations [27]. These findings highlight the clinical importance of the tumor site where CPS is evaluated, as certain sites show higher PD-L1 positivity rates and may therefore be preferred for biopsy to assess CPS.

Substantial evidence indicates that up to 50% of patients exhibit PD-L1 discordance between matched primary tumors and distant metastases [28]. Of note, pembrolizumab is approved for use in advanced TNBC with CPS ≥ 10 in either primary breast or metastatic tissue, as per the KEYNOTE-355 protocol [8]. However, the clinical implications of administering pembrolizumab to patients with discordant PD-L1 expression across different sites remain unclear. In addition, patients whose primary tumors were initially PD-L1 negative but who develop PD-L1 positive metastases demonstrate improved OS, highlighting the critical role of re-biopsy upon recurrence [28]. Despite the potential to expand therapeutic eligibility, re-biopsy at the time of progression has not yet been widely adopted in standard clinical practice.

While we did not evaluate CPS in metastatic tissues and thus could not replicate these findings, we observed that PD-L1 positivity was higher in pre-treatment surgical specimens compared to biopsy samples across both established clinical thresholds. Specifically, surgical specimens demonstrated nearly double the positivity rates of initial core needle biopsies, a finding that carries profound clinical implications for biomarker determination. Studies using the 22C3 pharmDx assay have reported discordance rates of approximately 28% between biopsies and surgical specimens, indicating that PD-L1 can be negative in biopsies but positive in surgical specimens or vice versa [29]. Similar findings have been reported with the SP142 assay, where up to one-third of cases negative on core biopsies were positive in surgical specimens [30]. From a biological perspective, this discrepancy is probably driven by intra-tumoral heterogeneity and sampling bias. PD-L1 expression in TNBC is rarely uniform; rather, it is highly localized and often concentrated at the invasive tumor front or within specific immune niches. A standard core needle biopsy only captures a narrow, linear track of tissue, creating an inherent sampling bias that can easily miss focal areas of high expression. Conversely, a surgical resection provides an extensive surface area that allows the central pathologist to evaluate multiple tumor blocks and geographic areas, significantly increasing the probability of capturing patches of robust CPS expression.

Beyond tumor biology, critical technical and pre-analytical variables likely contributed to these differences. Diagnostic core needle biopsies are frequently obtained in outpatient clinics where pre-analytical parameters, such as the exact time to fixation or the total duration of immersion in buffered formalin, are less tightly controlled. Delayed fixation or over-fixation can lead to masked epitopes and artifactual loss of antigenicity, artificially lowering the CPS. In contrast, surgical specimens at our reference institutions follow highly standardized processing pathways with optimized cold ischemia times, preserving protein expression far more reliably. Consequently, these findings highlight that it is essential not to rely solely on initial negative biopsy results; surgical specimens should also be evaluated to avoid excluding potentially PD-L1 positive cases from receiving pembrolizumab in the metastatic setting, especially when the evaluation is performed on archival primary tumor instead of metastatic tissue.

The clinical applicability of our findings carries profound implications for the structure of oncology care and patient access within the Mexican healthcare system. In Mexico, universal public coverage for premium targeted agents and immune checkpoint inhibitors remains severely constrained, meaning that the introduction of therapies like pembrolizumab frequently inflicts substantial financial toxicity on patients and their families. This access gap is further exacerbated by technical and infrastructural barriers, as standardized companion diagnostics, such as the 22C3 pharmDx automated assay, are not universally available across all regional hospitals and are typically restricted to centralized academic reference centers or private pathology networks. Consequently, many patients face prolonged diagnostic delays or are never tested due to logistical and financial hurdles. Our finding that baseline pre-treatment surgical specimens yield significantly higher rates of CPS expression compared to initial core needle biopsies offers a highly practical, resource-efficient strategy for resource-limited settings. By prioritizing the systematic evaluation of larger surgical specimens, clinicians can maximize the detection of treatment-eligible candidates without the need for performing repetitive, invasive, and costly re-biopsies. Addressing these systemic gaps in testing infrastructure and public procurement is essential to ensure that the clinical benefits of biomarker-driven immunotherapy can be equitably distributed across the unselected Mexican population.

Despite the valuable insights gained from this study, several limitations should be noted. First, this was a retrospective analysis, which inherently carries potential biases related to patient selection and missing data. Specifically, the requirement for available and sufficient archival formalin-fixed paraffin-embedded tissue blocks may have introduced a selection bias, as patients with very low tumor volume or those who only underwent fine-needle aspiration could be underrepresented. While the precise number of otherwise eligible patients who lacked available blocks across the fifteen-year period could not be quantified retrospectively, this challenge reflects a common limitation in real-world biomarker studies utilizing archival tissue in middle-income countries. Additionally, because this study was designed as a descriptive prevalence mapping of a regional cohort, all statistical associations were evaluated using unadjusted univariate models. We did not perform a multivariate adjusted analysis, as the strong biological interplay between advanced histological grade and dense immune infiltration, combined with the technical sampling variations between core biopsies and surgical resections, introduces significant collinearity that could destabilize independent regression models.

Furthermore, while we focused on PD-L1 expression using the 22C3 pharmDx assay, our findings may only partially reflect PD-L1 expression detected by other assays, such as SP142, which could limit the generalizability of our results to studies using different methodologies. Finally, we could not evaluate PD-L1 expression in metastatic tissues, as CPS assessments were limited to primary tumors, restricting our ability to thoroughly explore PD-L1 discordance between primary and metastatic sites. The lack of metastatic tissue evaluation also limits our ability to make definitive conclusions about how PD-L1 expression changes with disease progression.

## 5. Conclusions

In conclusion, our findings underscore the significant variability in PD-L1 expression across different clinicopathological features and TNBC tissue types. The strong association between higher histological grades, increased TILs, and PD-L1 positivity highlights the critical role of the immune microenvironment, particularly in more aggressive TNBC tumors. Importantly, the substantial difference in CPS positivity rates observed between baseline core needle biopsies and larger surgical specimens emphasizes that tissue volume and sampling methods significantly influence biomarker determination. This disparity supports a practical, resource-efficient clinical strategy for resource-limited settings: the larger pre-treatment surgical resection specimen should be prioritized to maximize the identification of treatment-eligible candidates. While our local data are limited to primary tissue, the established external literature indicates that PD-L1 expression can evolve throughout the disease course, and re-biopsy should be considered at recurrence or progression to optimize immunotherapy selection.

Future research should focus on understanding the biological implications of PD-L1 as a biomarker, including the differences between native and treatment-induced PD-L1 expression. Additionally, more studies are needed to explore PD-L1 positivity rates in larger and more diverse population cohorts, to improve personalized treatment strategies for TNBC patients. This will refine patient selection for immunotherapy and enhance our understanding of PD-L1’s role in TNBC progression and response to treatment.

## Figures and Tables

**Table 1:** PD-L1 expression by CPS across various parameters in a Mexican cohort of women with triple-negative breast cancer. Bold number means statistically significant.

	PD-L1 Expression by CPS					
	<1	≥1	*p*-Value	<10	≥10	*p*-Value
***n* (%)**	202 (70.9%)	83 (29.1%)	-	247 (82.9%)	38 (13.3%)	-
**Age (mean ± standard deviation)**	49 ± 12	48 ± 12	0.681	50 ± 12	44 ± 11	**0.008**
**Obesity (body mass index ≥ 30 kg/m^2^)**			0.208			0.746
No	142 (70.3%)	52 (62.7%)		169 (68.4%)	25 (65.8%)	
Yes	60 (29.7%)	31 (37.3%)		78 (31.6%)	13 (34.2%)	
**Histological type**			0.143			0.750
Ductal	182 (90.5%)	79 (95.2%)		225 (91.5%)	36 (94.7%)	
Non-ductal	19 (9.5%)	4 (4.8%)		21 (8.5%)	2 (5.3%)	
**Histological grade**			**0.035**			0.437
1	4 (2.1%)	0		4 (1.7%)	0	
2	37 (19.4%)	7 (8.8%)		40 (17.1%)	4 (10.8%)	
3	150 (78.5%)	73 (91.3%)		190 (81.2%)	33 (89.2%)	
**Ki-67**			0.374			0.854
<20%	26 (16.7%)	9 (12.2%)		29 (15.0%)	6 (16.2%)	
≥20%	130 (83.3%)	65 (87.8%)		164 (85.0%)	31 (83.8%)	
**Tumor-infiltrating lymphocytes**			**0.007**			**<0.001**
<30%	180 (90.0%)	63 (77.8%)		219 (89.4%)	24 (66.7%)	
≥30%	20 (10.0%)	18 (22.2%)		26 (10.6%)	12 (33.3%)	
**Tissue type**			**<0.001**			**<0.001**
Biopsy	140 (69.3%)	36 (43.4%)		164 (66.4%)	12 (31.6%)	
Surgical specimen	62 (30.7%)	47 (56.6%)		83 (33.6%)	26 (68.4%)	
**Time elapsed between biopsy/surgery and CPS evaluation**			0.258			0.479
<10 years	38 (18.8%)	11 (13.3%)		44 (17.8%)	5 (13.2%)	
>10 years	164 (81.2%)	72 (86.7%)		203 (82.2%)	33 (86.8%)	
**Lymph node status**			0.249			0.633
Negative	48 (23.9%)	25 (30.5%)		62 (25.3%)	11 (28.9%)	
Positive	153 (76.1%)	57 (69.5%)		183 (74.7%)	27 (71.1%)	
**Clinical stage (AJCC 8th edition)**			0.519			0.881
I	11 (5.4%)	3 (3.6%)		12 (4.9%)	2 (5.3%)	
II	62 (30.7%)	31 (37.3%)		79 (32.0%)	14 (36.8%)	
III	91 (45.0%)	38 (45.8%)		112 (45.3%)	17 (44.7%)	
IV (de novo)	38 (18.8%)	11 (13.3%)		44 (17.8%)	5 (13.2%)	
**Disease setting**			0.258			0.479
Early	164 (81.2%)	72 (86.7%)		203 (82.2%)	33 (86.8%)	
Metastatic	38 (18.8%)	11 (13.3%)		44 (17.8%)	5 (13.2%)	
**Pathological complete response**			0.145			0.285
No	51 (67.1%)	15 (51.7%)		59 (64.8%)	7 (50.0%)	
Yes	25 (32.9%)	14 (48.3%)		32 (35.2%)	7 (50.0%)	

## Data Availability

The data presented in this study are available upon reasonable request from the corresponding authors.
